# Tough gel adhesive is an effective method for meniscal repair in a bovine cadaveric study

**DOI:** 10.1186/s40634-023-00691-z

**Published:** 2023-12-14

**Authors:** David Mazy, Christopher Chung-Tze-Cheong, Zhenwei Ma, Ran Huo, Stephanie Lamer, Jianyu Li, Marie-Lyne Nault

**Affiliations:** 1 University of Montreal, 2900 Boul. Edouard-Montpetit, Montréal, QC H3T 1J4 Canada; 2 CHU Sainte-Justine, 3175 Chemin de La Côte-Sainte-Catherine, Montréal, QC H3T 1C5 Canada; 3 Department of Mechanical Engineering, McGill University, 817 Sherbrooke Street West Montreal, Quebec, H3A 0C3 Canada; 4 Department of Biomedical Engineering, McGill University, 3775 Rue University Montréal, Montreal, QC H3A 2B4 Canada; 5 Department of Surgery, McGill University, 1650 Cedar Ave, Montreal, QC H3G 1A4 Canada; 6 Department of Orthopedic Surgery, CIUSSS Hôpital du Sacré-Coeur de Montréal (HSCM), 5400 Boul. Gouin Ouest, Montreal, QC H4J 1C5 Canada

**Keywords:** Meniscal repair, Meniscal suture, Tough gel adhesive, Hydrogel adhesive

## Abstract

**Purpose:**

To test tough gel adhesives to repair meniscus tears under relevant loading conditions and determine if they have adequate biomechanical properties to repair meniscus tears in a bovine cadaveric study.

**Methods:**

Cyclic compression tests on 24 dissected bovine knees were performed. The tough gel adhesive was used either as an adhesive patch or as a coating bonded onto commercially available surgical sutures. Forty-eight menisci were tested in this study; 24 complete radial tears and 24 bucket-handle tears. After preconditioning, the specimens underwent 100 cycles of compression, (800 N/0.5 Hz) on an Instron© machine and the size of the gaps measured. One third of the menisci were repaired with pristine sutures, one third with adhesive patches, and one third with sutures coated in adhesive gel. The size of the gaps was compared after 100 and 500 cycles of compression.

**Results:**

The mean gap measured at the tear site without treatment was 6.46 mm (± 1.41 mm) for radial tears and 1.92 mm (± 0.65 mm) for bucket-handle tears. After treatment and 500 cycles of compression, the mean gap was 1.63 mm (± 1.41 mm) for pristine sutures, 1.50 mm (± 1.16 mm) for adhesive sutures and 2.06 mm (± 1.53 mm) for adhesive gel patches. There was no significant difference between treatments regardless of the type of tear. Also, the gaps for radial tears increased significantly with the number of compression cycles applied (*p* > 0.001).

**Conclusion:**

From a biomechanical standpoint, the tough adhesive gel patch is as effective as suturing. In addition, it would allow the repair of non-suturable tears and thus broaden the indications for meniscus repair.

**Level of evidence:**

Controlled laboratory study.

## Introduction

Historically, the preferred treatment for acute and unstable meniscus injuries has been meniscectomy, which involves partial or complete removal of the meniscus depending on the tear. However, there has been a paradigm shift over the years once the importance of preserving as much meniscal tissue as possible became paramount, to avoid early-onset osteoarthritis caused by meniscectomy [1, 2]. Hence, meniscal repairs are gaining ground in knee surgery [3, 4].

Meniscal repair has its own limitations. First, not all meniscus injuries are repairable, and success is not guaranteed [5, 6]. Studies report between 60 and 90% good clinical outcomes in the medium and long term, but much depends on the type and location of the tear [7–9]. Suturing the meniscus is also more time consuming and technically challenging than a meniscectomy [6, 9]. There is also a risk of complications such as neurovascular injuries [10]. Although new all-inside fixation methods could mitigate these complications [11, 12], the rate of secondary meniscectomy remains between 15 and 24% [13]. Nonetheless, meniscal repair plays a key role in knee surgery, calling for new technologies to improve outcomes [14].

Tissue adhesives are now used in thoracic surgery, neurosurgery, and plastic surgery [15]. However, in orthopedics, they are mainly used to limit blood loss in arthroplasties, for the treatment of osteochondral lesions, or as an additive to tendon and ligament repairs [16, 17]. With the advent of new biomaterials, attempts have been made to engineer hydrogels and tissue adhesives for meniscal repair, [18–20] which have been tested in vitro and in vivo [21–23]. Tough hydrogel adhesives offer new possibilities for meniscal repair especially as adhesion is possible in saline environments [24]. However, none of the adhesives available are adapted to the meniscus [25]. Indeed, a systematic review highlighted that the composition, vascularization and biomechanics of the meniscus, as well as the technical constraints associated with arthroscopy made the tissue adhesives currently used unsuitable [25–27]. Specifically, the meniscus bears a huge mechanical load, contains few cells, and has a blood supply that tends to decrease with age and is mostly peripheral which makes healing very complex [28]. Moreover, arthroscopies are performed in a very tight space and generally with a continuous flow of fluid [29].

In this study, we will use an ex vivo bovine knee model exposed to cyclic compressive loading and assess the performance of the tough gel adhesive to treat both radial and bucket-handle meniscus tears. A simple suture will be compared with a tough gel adhesive-coated suture and a tough gel adhesive patch. The aim of this study is to test tough gel adhesives to repair meniscus tears under relevant loading conditions and determine if they have the right biomechanical properties to repair meniscus injuries.

Our hypothesis is that the tough gel adhesive has similar biomechanical properties to meniscal sutures in maintaining the edges of a meniscal tear in this ex-vivo bovine model.

## Materials and methods

### Preparation of adhesives

A) Adhesive Gel patch. The synthesis protocol was the same as reported previously [30]. Briefly, the gel was produced by mixing acrylamide (AAm) of synthetic origin and alginate of natural origin. Ammonium persulfate (APS) was added, followed by methylene bisacrylamide (MBAA) to act as a crosslinker. Calcium sulphate (CaSO_4_) was used to crosslink the alginate chains. Finally, tetramethyl-ethylenediamine (TEMED), was added and the mixture polymerized overnight. Afterwards, the 1 mm thick patch was cut to a standard dimension of 50 mm long by 20 mm wide. The optimal size was determined with pilot tests on bovine menisci treated for radial and bucket-handle tears. Prior to patch application, 0.5 ml of 2% Chitosan mixed with 1-ethyl-3-(-3-dimethylaminopropyl) carbodiimide hydrochloride (EDC) and N-hydroxysuccinimide (NHS) was applied between the patch and the meniscal tissue. This substance is the cross-linker and activates adhesion. 0.5 ml ensured sufficient adhesion while minimizing leakage at the edges of the patch, which would lead to unwanted bonding with neighbouring structures.

B) Adhesive Sutures. The modified sutures comprised three major components: 1) a strong suture 2) a dehydrated tough gel sheath and 3) an adhesive reagent solution. For the suture, Vicryl® 2–0 was used. Following a previously reported technique [31], the coating process included pre-treatment of the sutures with NaOH, followed by a thorough wash with deionized water before air drying. The sutures were then soaked for 10 min in a mixture of chitosan, EDC, and NHS in a capillary tube. To form the gel sheath, the prepolymer solution was injected into the capillary tube to replace the adhesive solution. After one hour, the adhesive sutures were removed from the tube and immersed in CaCl_2_ solution for at least 10 min before use or left indefinitely for storage. The sutures were then air dried to dehydrate the gel sheath. Before suturing, 1 ml of a mixture of 2% Chitosan, EDC, and NHS was applied for rehydration and adhesion formation. This allowed the suture to expand and adhere to the tissue.

### Characteristics and preparation of bovine knees for testing (Fig. 1)

**Fig. 1:**
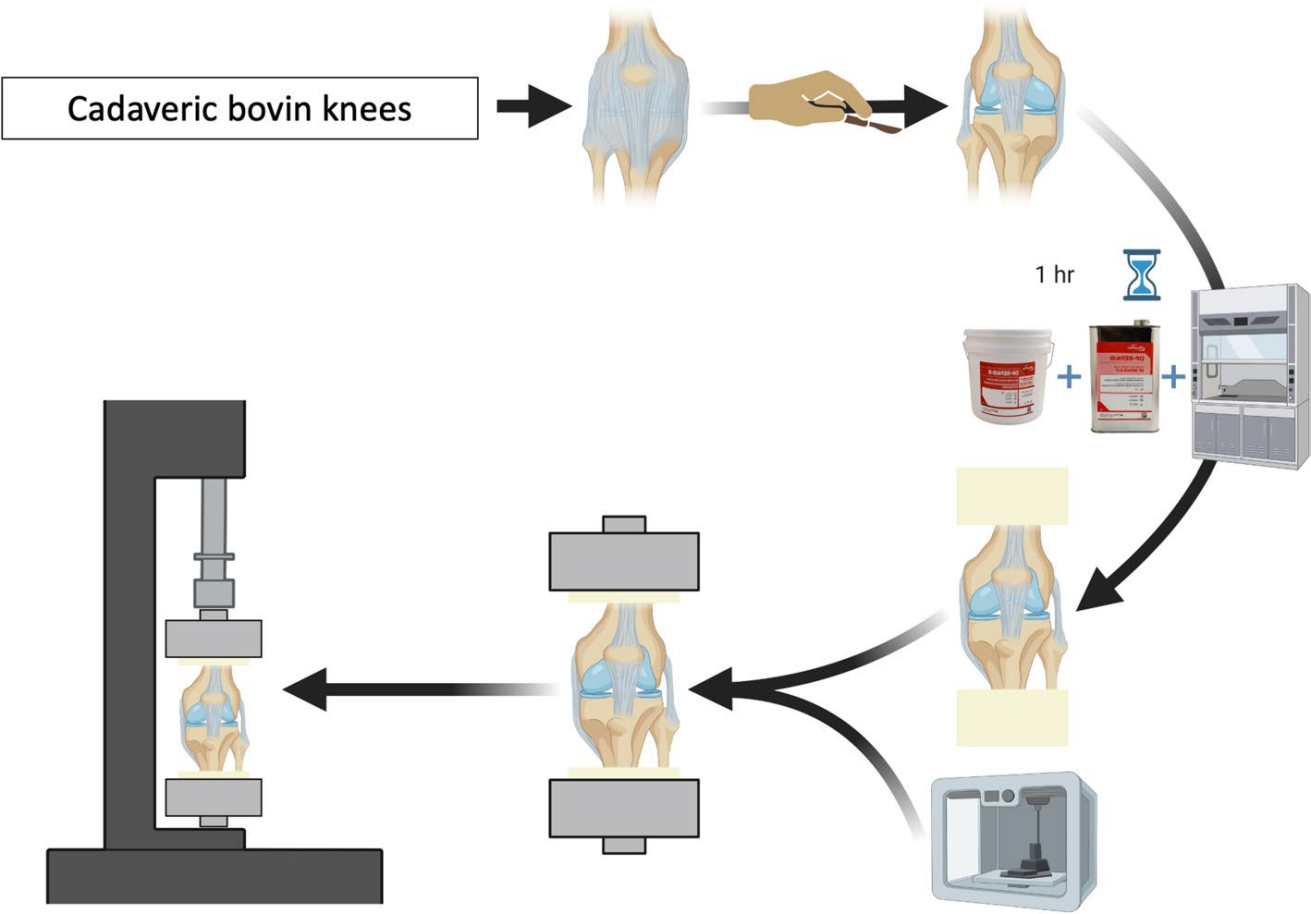
Methods for cadaveric knees. From dissection, through resin fixation and 3D-printed attachment device development, to installation on Instron machine

Cadaveric bovine knees were used for this study. There was no evidence of osteoarthritis, ligamentous damage, or meniscal damage on any of the specimens. There was no animal sacrifice for this study and ethics approval was not needed since all specimens came from the food industry. Specimens were removed from the -70° Celsius freezer at least 24 h before manipulation and dissection. Twenty-four cadaveric bovine knees were dissected, giving us access to the corresponding 48 menisci. The femur was cut approximately 20 cm (cm) from the joint to ensure secure diaphyseal fixation. The tibia and fibula were cut about 3 cm from the joint to provide a wider base and account for the inverted pyramidal shape of the tibia. The cruciate ligaments, collateral ligaments, intermeniscal ligament, meniscofemoral ligament, and meniscotibial ligaments were retained in order to preserve knee stability. The muscle bodies and extensor system were removed for better access to the menisci. Since the tests were performed in pure compression without knee flexion, the patella and extensor system were not needed. The ends of the bone were fixed in a resin made of DP-REPAIR liquid and DP-REPAIR-R Powder (DenPlus®) self-curing acrylic resin. This made it possible to standardize the templates for the bone extremities and fixation to the testing apparatus. We used 3D printed material to connect the various components to the testing device. The bovine tissue was hydrated with saline throughout the procedure to prevent drying.

### Meniscus tears

Twenty-four complete radial tears, up to the capsular attachment, were made on the lateral menisci where the anterior and middle third meet. Twenty-four bucket-handle tears were made on the medial menisci, starting 5 mm (mm) from the anterior horn to 5 mm before the posterior horn. To confirm that the tear was a bucket-handle, dislocation had to be in the inter-condylar notch. These bucket-handle tears were reduced, using surgical tools, before treatment. All radial tears were performed with a n°11 scalpel blade. Bucket-handle tears were started anteriorly with the same scalpel and completed posteriorly with curved scissors. All surgeries were performed with an open approach.

### Treatments applied

**Fig. 2:**
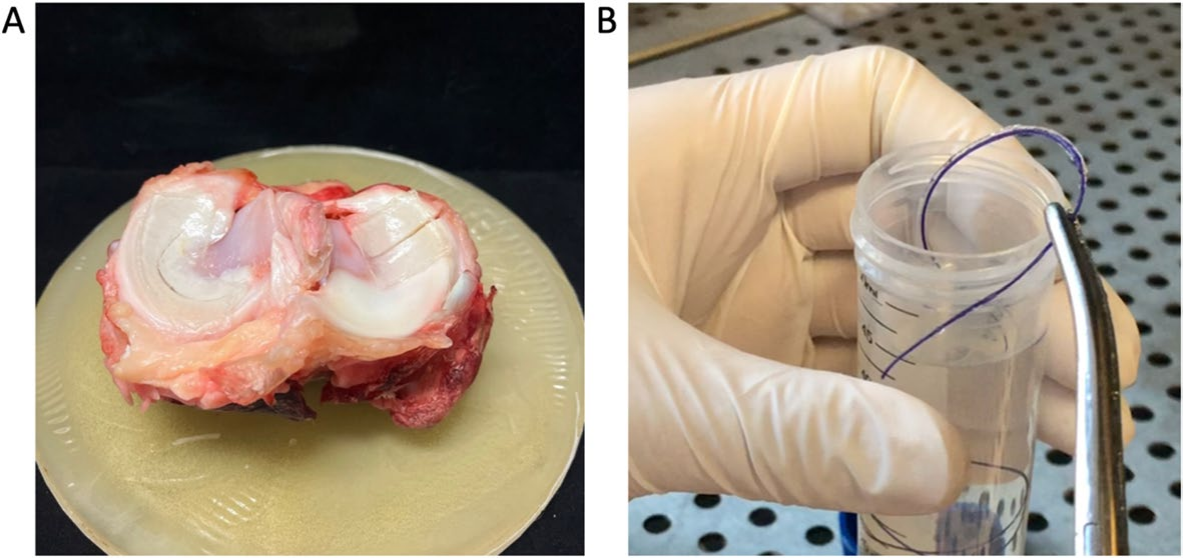
**A** Gel patch on a bucket-handle tear (left) and on a complete radial tear (right). **B** Ready-to-use adhesive suture

**Fig. 3:**
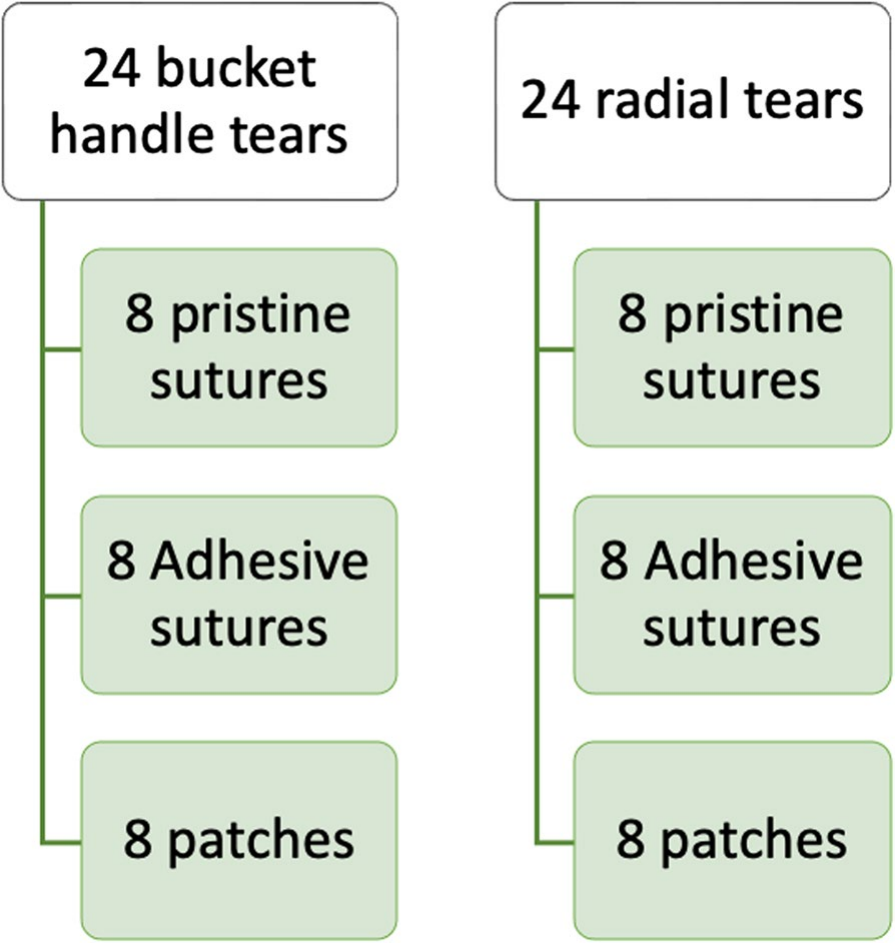
Group distribution

One third of the tears were repaired with a Vicryl® (polyglactin910) 2–0 suture, hereafter referred to as pristine sutures. Another third were repaired with a Vicryl® 2–0 suture coated with an adhesive gel, hereafter referred to as adhesive sutures. Finally, the remaining third were repaired with an adhesive gel patch (Figs. 2 and 3). The sutures were made with a FIRSTPASS © MINI (Smith&Nephew®). One suture was made in the center of the tear at 5 mm from each edge. The sutures consisted of a double knot and then two single inverted knots. The gel patches were cut to a standard size of 20 mm wide and 50 mm long. An adhesive solution was spread on the side of the patch in contact with the meniscus. The patch was then placed on the upper surface of the meniscus for bucket-handle tears. For radial tears, the patch was wrapped around the meniscus starting from the lower surface to the upper surface, passing through the central side. The patch was then compressed on the meniscus for 2 min for proper adhesion between the patch and the meniscal tissue. One suture was made in the center of the tear. In terms of adhesion, our hydrogel features an interfacial bond, which is a type of mechanical interlock [31]. It provides a physical barrier to the propagation of fissures at the interface. It also serves to increase the surface area, thereby increasing the total contact between adhesive and substrate. Adhesion is not chemical, but mechanical.

### Biomechanical testing

**Fig. 4:**
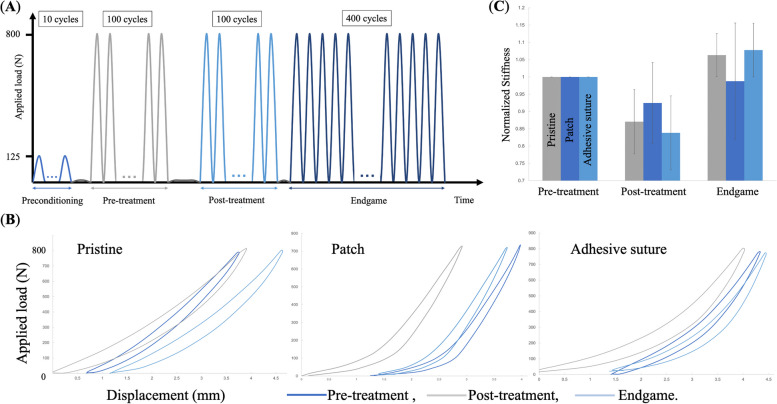
Mechanical assessment of whole bovine knees where menisci are treated with pristine sutures, patches or adhesive sutures. **A** Cyclic loading profile in four phases: preconditioning (10 cycles), pre-treatment (100 cycles), post-treatment (100 cycles) and endgame (400 cycles). **B** Force–displacement curves for the specific cycles indicated are plotted for three treatment conditions. **C** Normalized stiffness of repaired menisci in post-treatment and endgame

The entire knee joints were placed in full extension on the Instron 5965 (Instron Inc, Norwood, Massachusetts), and then subjected to cyclic mechanical loading **(**Fig. 1**)**. The mechanical loading profile mimicked physiological conditions as illustrated in Fig. 4A. Specifically, a preconditioning of 10 cycles around 125 Newtons (N) at a frequency of 0.5 Hz was performed. The tears, initially left untreated, were compressed during 100 cycles between 0 N and ~ 800 N at a frequency of 0.5 Hz. The size of the gap between the meniscal edges, at the center of the tear, was measured using an electronic caliper. The tears were then repaired. Once the knees were treated testing resumed for 100 cycles of compression at ~ 800 N and a frequency of 0.5 Hz. The size of the gap was then measured in the same location as before. To reach a total of 500 cycles, 400 more cycles of compression under the same conditions were completed. The resulting gap was measured again. In case of suture failure or rupture, the failure mechanism was noted. Figure 4B shows load–displacement curves, from which the tangents of the linear portions were fitted for knee stiffness. The knee stiffness of treated meniscus (post-treatment and endgame) was further normalized by that of intact meniscus pre-treatment for comparison in Fig. 4C.

### Statistical analyses

SPSS Statistics software (version 28.0.1.0; IBM Corp, Armonk, NY, USA) was used for our statistical analyses. The differences between the pre-treatment and post-treatment gaps were compared for each group. A one-factor within-subject (compression 100 and 500 cycles) and one-factor between-subject (treatment) repeated measures analysis of variance was used for the two types of tears (radial or bucket-handle). In case of a significant treatment effect, 2:2 comparisons (post-hoc) were made between the groups, using a Bonferroni correction. To test the assumption of normality, sensitivity analyses were performed using non-parametric Kruskal–Wallis tests, given the sample sizes. A value of *p* < 0.05 was considered statistically significant.

## Results

**Fig. 5:**
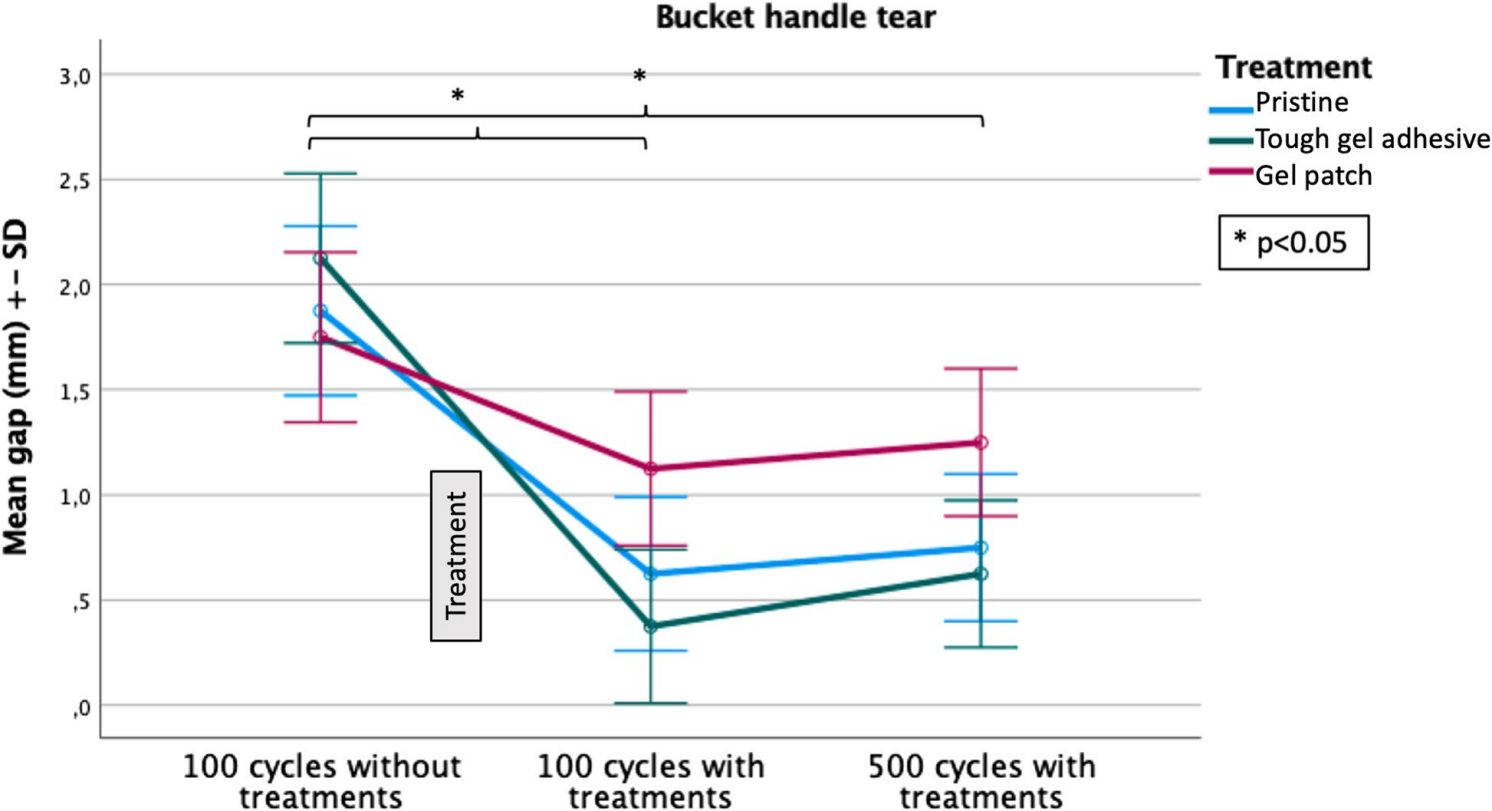
This figure concerns bucket handle tears. It compares the gap formation (mean gap (mm) ± standard deviation (SD)) after 100 cycles of compression without treatment and after 100 and 500 cycles of compression with treatment for each therapeutic group (Pristine, tough gel adhesive and gel patch). * indicates a significant difference. *N* = 8 for each group

**Fig. 6:**
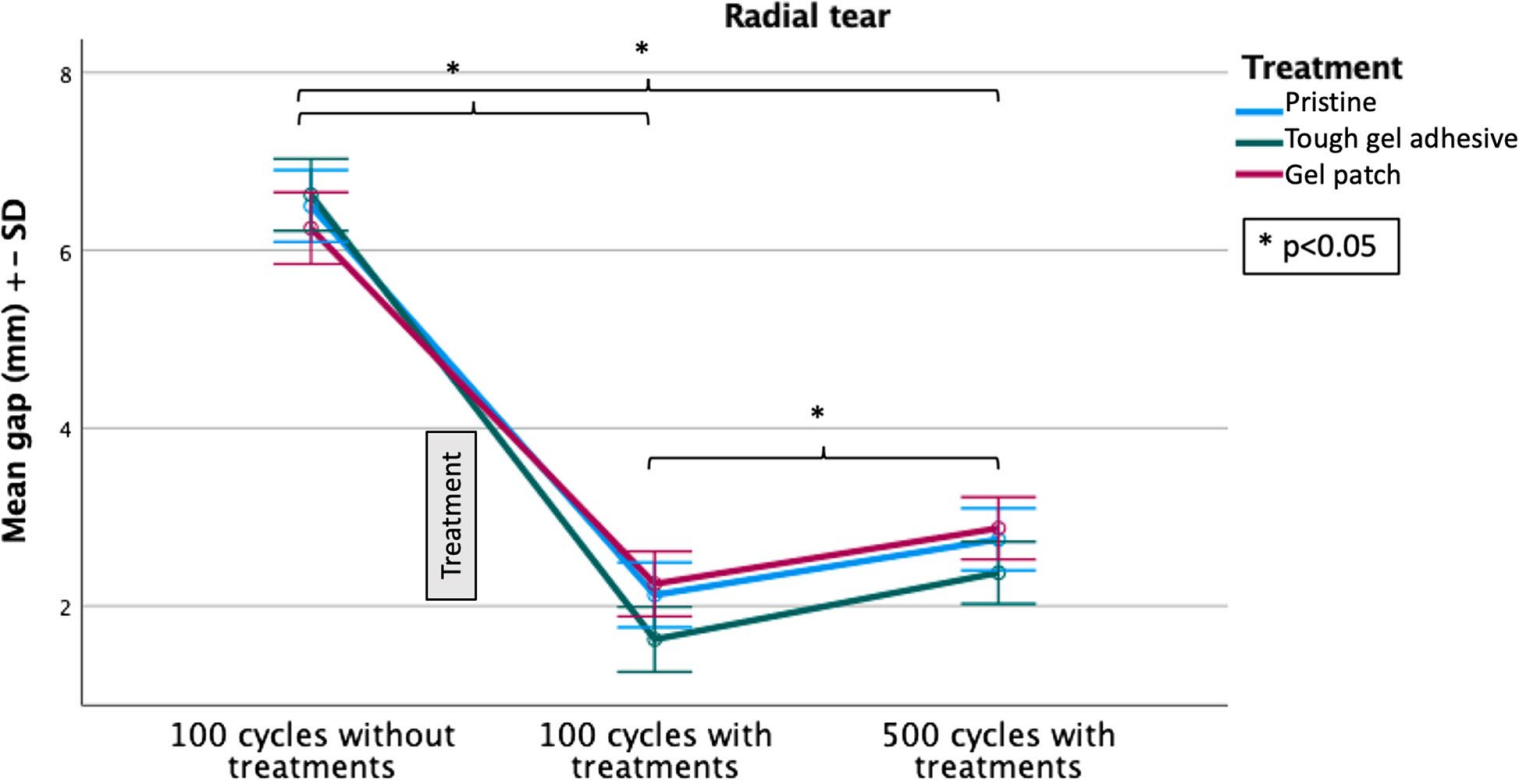
This figure concerns radial tears. It compares the gap formation (mean gap (mm) ± standard deviation (SD)) after 100 cycles of compression without treatment and after 100 and 500 cycles of compression with treatment for each therapeutic group (Pristine, tough gel adhesive and gel patch). * indicates a significant difference. *N* = 8 for each group

Table 1 lists the average size of the gaps (mm) according to the tear type. The mean gap measured at the tear site was 6.46 mm (± 1.41 mm) for radial tears and 1.92 mm (± 0.65 mm) for bucket-handle tears without any treatments. After treatment and 100 cycles of compression, the mean gap for radial tears was 2 mm (± 1.29 mm) and 0.71 mm (± 0.21 mm) for the bucket-handle tears. When the number of cycles is increased to 500 cycles, the gap formed increases to 2.67 mm (± 1.20 mm) for radial tears and to 0.88 mm (± 0.20 mm) for the bucket-handle tears. With treatment, gap is significantly reduced at 100 and 500 compression cycles (*p* < 0.001). This observation is valid for both radial and bucket-handle tears (Figs. 5 and 6).

**Table 1:** Comparison of the mean gap formation in millimeters ± standard deviation (SD) depending of the type of tear (radial or bucket handle). The comparison is made at 3 different times: after 100 cycles of compression without treatment, after 100 cycles and 500 cycles of compression with treatment

	**Number of tears**	**Gap after 100 cycles without treatment (mean** ± **SD)**	**Gap after 100 cycles with treatment (mean ± SD)**	**Gap after 500 cycles with treatment (mean ± SD)**
**Radial tears**	24	6.46 (± 1.41)	2 (± 1.29)	2.67(± 1.20)
**Bucket-handle tears**	24	1.92 (± 0.23)	0.71 (± 0.21)	0.88 (± 0.20)
Student’s t-test			***p* < 0.001**	***p* < 0.001**

Repeated measures analysis of variance shows that gap formation for bucket-handle tears was independent from the number of compression cycles (*p* = n.s.). For treated radial tears, the number of cycles had an impact on the size of the gap; it was significantly (*p* < 0.001) greater after 500 cycles than after 100 cycles (Fig. 6).

At 100 and 500 compression cycles, the post-hoc comparison of treatments, using a Bonferroni correction, did not show any significant differences between treatments (*p* = n.s.), both for radial and bucket-handle tears. This was applicable when comparing between pristine suture and adhesive suture, between pristine suture and adhesive patch, and between adhesive suture and adhesive patch. The statistical analyses are presented in Table 2. Normality tests also showed that there was no significant difference (*p* = n.s.) between the different treatments (Fig. 4C).

**Table 2:** Comparison of mean gap in millimeters (± SD) and corresponding *p*-values for subgroup analysis according to the type of tear

Bucket-handle			Radial		
	**Mean ± SD**	***p*-value**		**Mean ± SD**	***p*-value**
**100 cycles of compression with treatment**					
Pristine vs adhesive suture	0.6 ± 0.5 vs 0.4 ± 0.5	0.401	Pristine vs adhesive suture	2.1 ± 1.4 vs 1.6 ± 0.9	0.700
Pristine vs patch	0.6 ± 0.5 vs 1.1 ± 0.8	0.237	Pristine vs patch	2.1 ± 1.4 vs 2.3 ± 1.6	0.902
Adhesive suture vs patch	0.4 ± 0.5 vs 1.1 ± 0.8	0.462	Adhesive suture vs patch	1.6 ± 0.9 vs 2.3 ± 1.6	0.436
**500 cycles of compression with treatment**					
Pristine vs adhesive suture	0.8 ± 0.5 vs 0.6 ± 0.5	0.865	Pristine vs adhesive suture	2.8 ± 1 vs 2.4 ± 0.7	0.824
Pristine vs patch	0.8 ± 0.5 vs 1.3 ± 0.7	0.954	Pristine vs patch	2.8 ± 1 vs 2.9 ± 1.7	0.923
Adhesive suture vs patch	0.6 ± 0.5 vs 1.3 ± 0.7	0.640	Adhesive suture vs patch	2.4 ± 0.7 vs 2.9 ± 1.7	0.532

Sensitivity analyses using non-parametric tests validated our assumption of normality for bucket-handle tears (*p* = 0.065) and radial tears (*p* = 0.079).

Furthermore, all sutures and patches were intact after the different tests. There was no suture failure or suture penetration through the meniscal tissue. The adhesive gel coating process takes approximately 2 h, and there were no technical problems. The patch manufacturing process takes around 1 h, and no technical problems were observed.

## Discussion

The most important finding of the present study is that the tough gel adhesive showed comparable stability for meniscal repair as the conventional sutures in this cadaveric bovine study. In our biomechanical tests, the adhesive, used either as a coated suture or as an adhesive patch, was able to effectively maintain the edges of radial and bucket-handle tears together, while sustaining load. The gap formed at the tear sites was significantly smaller, regardless of treatment type. Overall, our results support the use of an adhesive patch in an animal cadaveric model for radial tears, which are difficult to treat, as well as longitudinal tears [32]. It is possible to infer that complex tears, usually subject to meniscectomy, could be treated with the adhesive patch [33]. Also, from a more clinical point of view, it is essential to maintain the edges of a lesion robustly to allow meniscal healing [34]. Although this study is purely biomechanical ex-vivo, and there is still a long way to go, it could serve as the basis for future studies that bring us closer to clinical applications.

In addition, this study refined the methods for the use of tough gel adhesives in meniscal repair. First, we determined that 0.5 ml of adhesive reagents (chitosan, EDC and NHS) was the most suitable during preliminary tests. With larger amounts, the reagent leaked from both sides of the patch, causing it to adhere in part to the femoral cartilage. It should be noted that the adhesive patch can be stored in a refrigerator for several months while maintaining its properties. Another interesting advantage of this adhesive hydrogel, composed of alginate and chitosan, is its manufacturing cost. Indeed, the cost of a 1 cm x 2 cm × 0.15 mm patch should not exceed 3 US dollars, including disposable materials and laboratory work. For this same patch, the cost of raw materials does not exceed one US dollar. Therefore, there is no comparison with intra-articular suture devices [35].

For the adhesive suture, we selected polyglactin910 (Vicryl) because of its strength and proven compatibility with tough gel adhesive coating. In theory, other sutures could also be used, as previous studies have shown [31]. The total preparation time for coated sutures is approximately 2 h, but they can be stored afterwards. However, between five and ten minutes are necessary for the coated suture to irreversibly bind with surrounding tissues. This is long enough to easily manage any modifications needed.

Tests on whole cadaveric bovine knees are relatively rare and this is the first biomechanical study to assess the performance of tough gel adhesive, in two forms, to treat meniscal tears. The biomechanical tests were designed to ensure physiological relevance and account for the testing capacity of our installation. As shown in Fig. 4, the loading profile includes cyclic compression of 800 N at 0.5 Hz for hundreds of cycles. The peak force was chosen to reflect the large size and quadrupedal nature of the bovine samples and because it is easy to achieve with our Instron machine (Model 5965, Norwood, Massachusetts). It should also be noted that there are a wide range of compression loads (300 N to 1000 N) reported in studies on human and animal menisci [36–39]. The frequency was chosen based on a typical working speed and its extensive use for cyclic tests in literature [32, 40]. Regarding the number of cycles and loading time, the purpose of this study was to compare the repair outcomes of different treatments, but not to test their long-term efficacy. As such, we included 100 cycles without treatment, to recreate the stress that our patients' knees might experience before treatment, 100 cycles with treatment for initial comparison, followed by another 500 cycles to assess failure rates or an increase in gap size. Admittedly, our tests were conducted within a much shorter time period than that required to heal menisci or to degrade Vicryl [41, 42]. Theoretically, the number of cycles can be much greater, but this is very time-consuming and demanding for the machine.

Our preliminary tests showed that complete radial and bucket-handle tears were needed to observe gap formation at the tear site and to assess the efficacy of different treatments. Specifically, when located exclusively in the avascular area, there was no gap formation for radial tears under our testing conditions, even without treatment; the same phenomenon was observed with longitudinal vertical tears of 10 mm or less. Our results echo those of a previous report stating that the meniscus responded very differently with a major radial tear (at least 90% complete) in terms of displacement and load transmission [43]. Also of interest, only radial tears responded differently to the number of compression cycles. When the number of cycles increased from 100 to 500, the gap for radial tears became significantly (*p* < 0.001) larger, whereas the number of cycles did not impact gap size for bucket-handle tears. These results reflect those found in the literature [44]. A likely cause is the fact that compression in a reduced bucket-handle tear brings its edges closer together, while separating them in radial tears [45, 46]. If the space formed was measured in complete compression, it is likely that this difference would be even greater, given the instability of radial lesions. This is the rationale behind studies that encourage early postoperative weight-bearing in longitudinal tears, such as bucket-handles, but not in radial tears [47]. These findings suggest that complex lesions, usually treated with a meniscectomy and representing up to 18% of meniscal injuries in pediatrics, could be treated with the adhesive patch using the meniscus wrapping method [48]. Adhesive hydrogels could represent a treatment method for tears in the red-red zone because of their mechanical edge-holding effect, but we believe that tears in the white-white zone could also be treated with the biological augmentation of the patch.

This study has several limitations. First, an open approach was used to install the patch, rather than the arthroscopic approach commonly used for meniscus repair [6, 49]. Second, the animal cadaveric model used here is different from the human meniscus. Given the complexity of the anatomy and forces involved, it is technically impossible to achieve the same mechanical loading conditions (local pressure) for animals and humans. Also, the tests were carried out in pure compression, and our machine did not allow for flexion and rotation tests. Nevertheless, the bovine knee model was chosen for its availability, affordable cost and extensive use in related research [50, 51]. Additionally, Vicryl is not the type of suture best suited for meniscal repair because of its degradation rate. However, since each individual test was conducted within one day, degradation could not become a significant variable. The Vicryl suture therefore retains its initial strength, comparable to that of sutures used in the clinic [41]. It should also be mentioned that the number of cycles would be much higher in an *in-vivo* model. This study cannot reveal the long-term and *in-vivo* intra-articular behavior of tough gel adhesives. To address these limitations, various studies are underway, including an arthroscopic feasibility study and an in-vivo animal study. In addition, a study of the patch's degradation rate in vitro is also in progress.

The promising results reported here support further research on tough gel adhesives for meniscal repair. Beyond the two kinds of meniscus tears tested here, the surgical indications for the adhesive patch could include non-suturable meniscus tears, when suturing is challenging but a patch might be feasible and beneficial. This is an important avenue to pursue. The adhesive patch also opens the door to hybrid models, where a suture could be enhanced by the addition of a patch, as studied in the treatment of massive rotator cuff tears [52]. The patch would facilitate a more even and effective distribution of stresses on the suture.

These efforts are expected to provide surgeons with a "meniscal toolbox", which would extend the surgical indications for meniscus repair and increase the procedure’s success rate. Accordingly, the long-term complications linked with meniscectomies, such as osteoarthritis, could be mitigated to reduce the socio-economic burden related to osteotomies or arthroplasties [2, 53].

## Conclusion

There is an increasing demand in orthopedic surgery for meniscal repair methods, while bioengineering and advances in the field of tissue adhesives offer new possibilities. From a biomechanical standpoint, the tough adhesive gel patch is as effective as suturing. In addition, it would allow the repair of non-suturable tears, thus broadening the indications for meniscus repair. Further work, especially in-vivo studies, is required to evaluate and develop these materials.

## Data Availability

Data available from the corresponding author upon reasonable request.
